# Fully Biobased Shape Memory Thermoplastic Vulcanizates from Poly(Lactic Acid) and Modified Natural *Eucommia Ulmoides* Gum with Co-Continuous Structure and Super Toughness

**DOI:** 10.3390/polym11122040

**Published:** 2019-12-09

**Authors:** Yan Wang, Jinhui Liu, Lin Xia, Mei Shen, Liping Wei, Zhenxiang Xin, Jinkuk Kim

**Affiliations:** 1 Key Laboratory of Rubber-Plastics, Ministry of Education/Shandong Provincial Key Laboratory of Rubber-Plastics, School of Polymer Science and Engineering, Qingdao University of Science and Technology, Qingdao 266042, China; m15092148968@163.com (Y.W.); liujinhui7@163.com (J.L.); 13954279735@163.com (L.X.); 02896@qust.edu.cn (M.S.); 18906423189@163.com (L.W.); 2 404-424 Elastomer Lab, Gyeongsang National University, 501 Jinju-daero, Jinju 52828, Korea; aikone71@163.com

**Keywords:** shape memory, poly(lactic acid), modified natural *Eucommia ulmoides* gum, dynamic vulcanization, in situ compatibilization, co-continuous structure

## Abstract

Novel, fully biobased shape memory thermoplastic vulcanizates (TPVs) were prepared using two sustainable biopolymers, poly(lactic acid) (PLA), and modified natural *Eucommia ulmoides* gum (EUG-*g*-GMA), via a dynamic vulcanization technique. Simultaneously, in situ compatibilization was achieved in the TPVs to improve interfacial adhesion and the crosslinked modified *Eucommia ulmoides* gum (EUG) was in “netlike” continuous state in the PLA matrix to form “sea-sea” phase structure. The promoted interface and co-continuous structure played critical roles in enhancing shape memory capacity and toughness of the TPVs. The TPV with 40 wt % modified EUG displayed the highest toughness with an impact strength of 54.8 kJ/m^2^ and the most excellent shape memory performances with a shape fixity ratio (*R_f_*) of 99.83% and a shape recovery ratio (*R_r_*) of 93.74%. The prepared shape memory TPVs would open up great potential applications in biobased shape memory materials for smart medical devices.

## 1. Introduction

Shape memory polymers (SMPs), a class of smart materials, have attracted much attention from academia and industry because they are able to change their shapes in response to environmental stimuli, such as heat [1], light [2], electricity [3], moisture [4,5], pH values [6], and magnetic fields [3]. In the past decade, researchers have made great progress in designing and fabricating SMPs, which grants the intelligent materials great potential applications in various fields, such as biomedical devices, smart sensors, self-healing materials, aerospace, and information carriers [7,8,9,10]. Of the described SMPs, researchers have investigated the heat-induced SMPs (HSMPs) extensively [1,11,12,13]. In general, there are two domains in HSMPs, reversible domain to freeze and unfreeze temporary shape and fixed domain to maintain permanent shape [14,15], and the two domains work in conjunction to achieve heat triggered shape memory effect (HSME). The mechanism for HSME can be described as follows. Firstly, the sample was stretched by a certain external force to the temporary shape above the transition temperature (*T*_trans_) [15,16], which would be glass transition temperature (*T*_g_) [17,18] or melting temperature (*T*_m_) [19] of the reversible phase. Then, the temporary shape was fixed by cooling to *T*_trans_, restricting the deformation and storing elastic force to the fixed domain for shape recovery. Finally, shape recovery was achieved by reheating to a temperature above *T*_trans_. Therefore, shape memory performance can be regulated through tailoring the microstructure of the two components [20,21].

Polymer blending of plastic and elastomer with merits of easy processing might be the most effective way to prepare HSMPs, in which the plastic phase served as a reversible domain and elastomeric phase was fixed domain [22]. A variety of shape memory thermoplastic vulcanizates (TPVs) such as PLLA/EVA TPVs [23,24], PLLA/PCL TPVs [25], PLLA/PMMA TPVs [26], have been developed based on the blending method, but low shape recovery capacity, unsustainability, and poor biocompatibility limited their further development and application, especially in biomedical fields.

It has been widely reported that the phase structure and interfacial adhesion of the rubber phase and plastic phase have a great influence on shape memory properties of the TPVs [27,28]. Therefore, changing the two parts in TPVs or altering their phase structure would result in tailored shape memory performances. Nevertheless, it is difficult to achieve strong interface interaction in most traditional rubber/plastic TPVs with typical “sea-island” phase structure because the two phases are usually immiscible [29,30]. Consequently, it is essential to improve the compatibility of the two components and achieve a co-continuous phase structure for promoting the shape memory capacity of TPVs.

Poly(lactic acid) (PLA), a kind of sustainable and degradable biopolymer, has been reported to exhibit HSME, however, inherent brittleness limited its application [31,32]. Moreover, natural *Eucommia ulmoides* gum (EUG) was also biocompatible and renewable as an isomer of natural rubber, which can be used as biobased HSMPs when it is partly crosslinked [14,33,34]. However, EUG is less compatible with PLA due to its non-polar molecular chains. In this effort, we firstly modified EUG by a bulk-free radical polymerization of glycidyl methacrylate (GMA) to introduce polar groups for improving its compatibility with PLA. Then the modified EUG was used with PLA to develop a new-typed shape memory TPVs with co-continuous phase structure, improved interface, and super toughness via an in situ dynamic vulcanization method. The prepared intelligent PLA/modified EUG TPVs would provide a new idea for the industrialization of smart materials.

## 2. Experimental Section

### 2.1. Materials

PLA, grade REVODE101, with average molecular weight (*M*_w_) = 1.5 × 10^4^ g/mol, *ρ* = 1.25 g/cm^3^, MFR (190 °C, 2.16 kg) = 5–8 g/10 min, was purchased from Zhengjiang Hisun Biomaterials Co Ltd. (Taizhou, China). EUG, (*M*_w_) = 1.8 × 10^5^ g/mol, PDI = 3.6, was provided by Hunan Xiangxilaodie Bio. Co Ltd. (Xiangxi, China). Dicumyl peroxide (DCP), recrystallized before use, was supplied by AkzoNobel Co Ltd. (Amsterdam, Holland). Glycidyl methacrylate (GMA) was purchased from Aladdin Co. Ltd (Shanghai, China). Pentaerythritol tetrakys 3-(3,5-ditert-butyl-4-hydroxyphenyl) propionate (antioxidant 1010) was supplied by Shandong Linyi Sanfeng Chemical Co., Ltd. (Linyi, China).

### 2.2. Preparation of Modified EUG

Natural EUG (153.0 g), GMA (27.0 g) and antioxidant 1010 (0.36 g) were mixed using a Haake Rheocord 90 chamber at 120 °C for 3 min. Then, grafting reaction of GMA to the EUG lasted 9 min after the addition of DCP (0.45 g) as initiator. The rotate speed was set to 60 r/min. Finally, the modified EUG was extracted by acetone for two days at 60 °C to remove the GMA monomer and GMA homopolymer (PGMA) and the residual EUG grafts (EUG-*g*-GMA) were dried in a vacuum oven at 30 °C for three days. The reaction yield of GMA was determined by the following equation [30].

(1) $$\mathrm{GMA}\;\mathrm{reaction}\;\mathrm{yield}=\frac{m_{\mathrm{GMA}}-m_{\left(\mathrm{PGMA}+\mathrm{GMA}\right)}}{m_{\mathrm{EUG}-g-\mathrm{GMA}}}\times 100\%$$

where the mass of GMA monomer was marked as *m*_GMA_, the mass of PGMA and residual GMA was named as *m*_(PGMA+GMA),_ *m*_EUG-*g*-GMA_ represented the mass of the modified EUG.

### 2.3. Preparation of the TPVs

The shape memory PLA/EUG-*g*-GMA TPVs and PLA/EUG TPV were compounded using a Haake Rheocord 90 at 135 °C with a rotate speed of 60 r/min. Firstly, PLA, EUG-*g*-GMA or EUG were added to the chamber for shear-melting until the torque was stable followed by the addition of DCP. Then, in situ dynamic vulcanization of the rubber phase was initiated by DCP and it took about 5 min for the torque to become stable again. Subsequently, the TPVs were hot-molded into sheets at 165 °C for 3 min under a pressure of 15 MPa followed by cold-pressing. Table 1 shows the formulas of the TPVs. For brevity, the sample codes for the TPVs were named according to their components and weight ratios. The PLA/EUG-*g*-GMA TPVs with weight ratios of 90/10, 80/20, 70/30, and 60/40 were named as P9EG1, P8EG2, P7EG3, and P6EG4, respectively, while the PLA/EUG TPV with a weight ratio of 60/40 was defined as P6E4.

The crosslinked EUG-*g*-GMA was prepared through a mechanical blending and chemical crosslinking method. Firstly, EUG-*g*-GMA lumps, antioxidant 1010 and DCP were added into the chamber for 5 min mixing. Then the blend was crosslinked at 165 °C for 10 min under a pressure of 15 MPa followed by cold-pressing.

### 2.4. Characterizations

#### 2.4.1. Fourier Transform Infrared Spectroscopy (FTIR)

A Bruker Tensor 27 spectrometer (Bruker, Bremen, Germany) was used to collect FTIR spectra of EUG, EUG-*g*-GMA, PLA and DCM-extracted PLA/EUG-*g*-GMA TPVs for three times. The attenuated total reflectance (ATR) mode was chosen. EUG, EUG-*g*-GMA, and PLA were tested directly, however, the PLA/EUG-*g*-GMA TPVs were extracted by DCM at 60 °C for three days, and the residuals were dried in a vacuum oven at 40 °C for three days before testing.

#### 2.4.2. Scanning Electron Microscopy (SEM)

Fracture appearance of neat PLA, PLA/EUG-*g*-GMA TPVs, and PLA/EUG TPV were observed using a JEOL JSM-6700 F field-emission SEM instrument (Japan Electronics Corp., Beijing, China) at an acceleration voltage of 5.0 kV. Before observation, the fractured samples were coated with platinum after being placed on an aluminum holder.

#### 2.4.3. Rheological Measurement

Rheological measurements of neat PLA and EUG-*g*-GMA at various temperatures (140, 150, 160 and 170 °C) were done by using ARES-G2 Rheometer (TA Company Boston, MA, USA). Frequency sweep was performed in a frequency range from 0.1 to 100 Hz, and the constant strain amplitude was 1%. Circle-shaped samples (diameter = 25 mm) were placed between the serration flat clamp.

#### 2.4.4. Different Scanning Calorimetry (DSC)

A DSC-Q2000 (TA Instruments, New Castle, DE, USA) was used to carry out thermal analysis for neat PLA, EUG-*g*-GMA and the TPVs. For all samples, three heating-cooling-heating cycles were performed from −60 to 180 °C in an N_2_ atmosphere. In each thermal cycle, the sample was maintained at 180 °C for 3 min to erase the thermal history before it was cooled to −60 °C. Then, the sample was heated to 180 °C subsequently. Both heating and cooling rate was 10 °C/min.

#### 2.4.5. Shape Memory Analysis

Shape memory analyses of crosslinked EUG-*g*-GMA, PLA, and PLA/EUG-*g*-GMA TPVs were performed on a DMA-Q800 instrument (TA Instrument, New Castle, DE, USA) based on the strain-controlled mode using rectangular-shaped specimens (width = 4 mm, thickness = 1 mm). The initial clamp gap was set to 4–6 mm and the preload was 0.001 N. Detailed procedures for shape memory analysis were as follows.

The sample was first kept isothermal at *T*_trans_ = 60 °C for 10 min to eliminate the stress history and the original strain was donated as ε_0_. Then, external stress was loaded to stretch the sample to the maximum strain of ε_1, load_ = 100% at a rate of 10%/min followed by a cooling process to −10 °C. The temporary shape with a strain of ε_1_ was fixed after unloading the stress and maintaining at −10 °C for 10 min. Finally, the sample recovered to its permanent shape with a strain of ε_0, rec_ as a result of being reheated to 60 °C. Both heating and cooling rates were 3 °C/min. Three shape memory cycles were performed for each sample.

To describe shape memory performances of neat PLA and the TPVs quantitatively, shape fixity ratio (*R_f_*), and shape recovery ratio (*R_r_*) were defined as follows [11], and they were calculated according to the first shape memory cycle.

(2) $$R_{f}=\frac{\varepsilon_{1}-\varepsilon_{0}}{\varepsilon_{1,load}-\varepsilon_{0}}\times 100\%$$

(3) $$R_{r}=\frac{\varepsilon_{1}-\varepsilon_{0,rec}}{\varepsilon_{1}-\varepsilon_{0}}\times 100\%$$

#### 2.4.6. Dynamic Mechanical Thermal Analysis (DMTA)

The DMA-Q800 instrument was also employed for DMTA characterization based on tensile mode using rectangular-shaped specimens (8 × 4 × 1 mm^3^). Temperature sweep was carried out from −95 to 100 °C at 3 °C/min. The constant frequency was set to be 10 Hz.

#### 2.4.7. Mechanical Property Measurements

Tensile behaviors of neat PLA and the TPVs were performed on a universal tensile testing machine according to ISO 527 with five dumbbell-shaped specimens at room temperature. The initial length of the specimen was 25 mm and the jaw separation was 500 mm/min. The stress-strain curves were recorded. 

According to the ISO 180, notched Izod impact performances of the TPVs at room temperature were conducted to further evaluate toughness using ten specimens. The impact energy and speed were 2.75 J and 3.50 m/s, respectively. The average impact strength was determined to be the notched Izod impact strength, and error bars were calculated using an STDEV formula to demonstrate the dispersion of the value relative to the mean.

## 3. Results and Discussion

### 3.1. Preparation of Modified EUG

**Figure 1:**
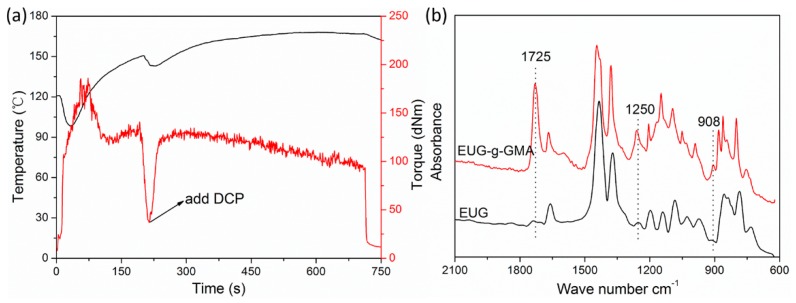
(**a**) Evolution of torque and temperature of *Eucommia ulmoides* gum (EUG) against time during modification, (**b**) Fourier transform infrared spectroscopy (FTIR) spectra of EUG before and after modification.

Given that EUG is less compatible with PLA, we modified EUG to introduce polar groups via the bulk radical grafting reaction of GMA monomer. The modification process was verified by the evolution of torque and temperature against time (Figure 1a). After mixing 200 s, the blend exhibited pretty high melting torque, and temperature increased gradually, which resulted from strong shear and friction during mixing. The torque increased abruptly after the addition of DCP, which might be attributed to the formation of EUG-GMA grafts and PGMA by self-polymerization of the GMA monomer. Then, the melting torque experienced a slow decrease after reaching the maximum value, which might be contributed by the continuing increase in temperature. To be noted, crosslinking and degradation of EUG and self-polymerization of the GMA might occur in such a modification process.

The modified EUG was extracted by acetone to remove residual GMA monomer and PGMA, and the reaction yield was determined to be 7.8%. Afterward, FTIR analysis further confirmed the grafting of GMA (Figure 1b). FTIR spectrum of the modified EUG was parallel to that of EUG with peaks at 1383 and 1666 cm^−1^, representing extension vibration of methyl groups and double carbon bonds (C=C), respectively. However, new absorption peaks occurred in the spectrum of modified EUG at 1725, 1250, and 908 cm^−1^, which were attributed to extension vibration of carbonyl (C=O) and epoxy groups. These new peaks, characteristic absorption of GMA, confirmed that a EUG graft (EUG-*g*-GMA) had been successfully prepared through the bulk free radical grafting process.

### 3.2. Preparation and Co-Continuous Structure in PLA/EUG-g-GMA TPVs

**Figure 2:**
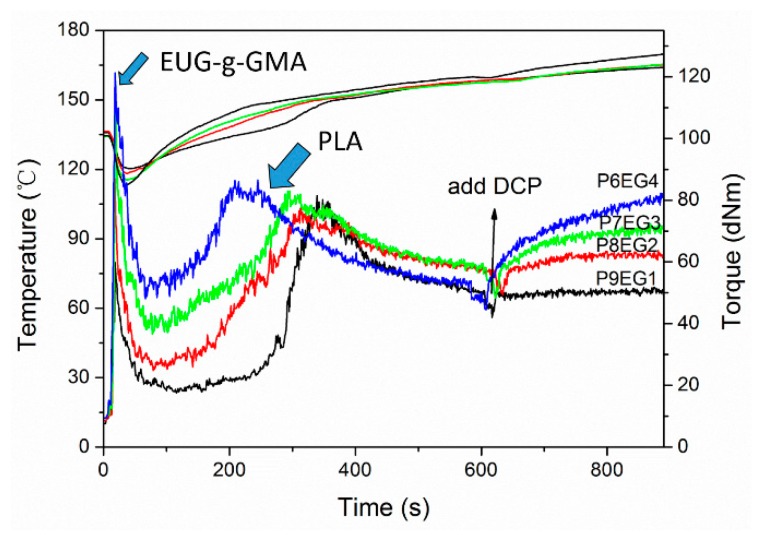
Changes in torque and temperature of the PLA/EUG-*g*-GMA blends during mixing.

The prepared EUG-*g*-GMA was used with PLA to develop fully biobased PLA/EUG-*g*-GMA TPVs via in situ dynamic vulcanization method and Figure 2 shows the changes in torque and temperature of the PLA/EUG-*g*-GMA blends during mixing. As we can see, there were two peaks in the torque curve within 600 s, which were correlated with the melting of EUG-*g*-GMA at a lower temperature and melting of PLA at a higher temperature. Though the peak values were different, they showed a linear relationship with the content of the two components. The melting peak for the PLA phase shifted to a lower temperature as EUG-*g*-GMA content increased, indicating that PLA was melted faster due to stronger shear and frication from the EUG-*g*-GMA phase. Before adding DCP, the first stable melting torque with a value of nearly 50 dNm was reached by any PLA/EUG-*g*-GMA blend, which demonstrated that the PLA phase was the dominator of melting viscosity before vulcanization of the rubber phase. After the addition of DCP, the melting torque firstly experienced a significant increase as an indicator of the vulcanization of the EUG-*g*-GMA phase and then achieved the second stable state to imply the accomplishment of dynamic vulcanization.

**Figure 3:**
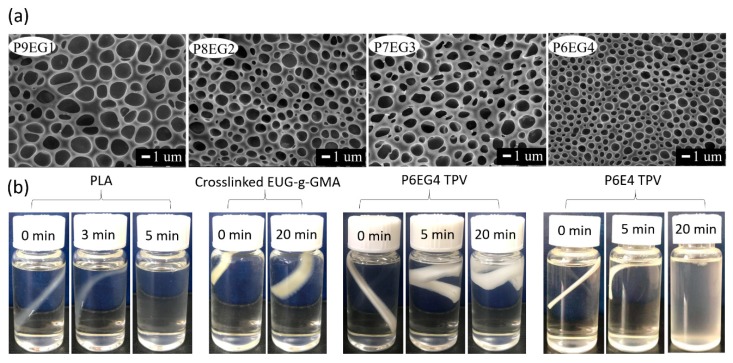
(**a**) SEM images of DCM-etched cryogenically fracture surfaces. (**b**) Digital photos of swelling experiments.

It has been widely reported that phase morphology plays a critical role in polymer blends and most traditional TPVs gave a typical “sea-island” phase structure. Herein, SEM was employed to observe the phase structure in the PLA/EUG-*g*-GMA TPVs. Before observation, the fractured TPVs were placed in DCM solvent for 3 min at room temperature in order to remove the unreacted PLA completely and the DCM-etched samples were used for SEM testing. The crosslinked EUG-*g*-GMA phase showed “netlike” structure in Figure 3a, which was quite different from the “sea-island” structure in traditional TPVs. The novel “sea-sea” structure was believed to make a contribution to enhancement in interfacial adhesion and final properties. Swelling experiments were designed to further prove the co-continuous structure. As shown in Figure 3b, PLA was dissolved in the DCM absolutely within 5 min, however, the crosslinked EUG-*g*-GMA was only swollen after 20 min. Moreover, the P6EG4 TPV was not dissolved but crimped without support from the dissolved PLA phase and the solution was still clear and transparent, which strongly indicated the continuous structure of crosslinked EUG-*g*-GMA phase. If the EUG-*g*-GMA was dispersed in the PLA matrix as particles, the DCM-etched residuals would be dispersed in the solvent to form suspension like the situation of P6E4 TPV shown in the figure.

**Figure 4:**
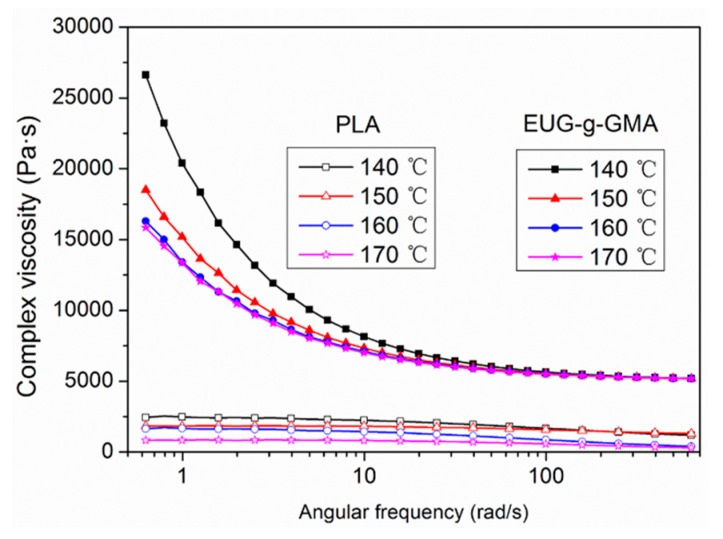
The dependence of complex viscosity of PLA and EUG-*g*-GMA on the frequency at various temperatures.

Since we have proved the co-continuous structure in the PLA/EUG-*g*-GMA TPVs, rheological behaviors of PLA matrix and EUG-*g*-GMA at various temperatures were studied to further investigate the essential formation mechanism of the phase morphology, shown in Figure 4. Generally, a minor component is ruptured to be dispersed domain during melting blending, forming the typical “sea-island” phase structure in polymer blends. Wu [35] proposed the following correlation to explain the formation mechanism of various phase morphology in polymer blends:

(4) $$d=\frac{{4\alpha K\;}^{\pm 0.84}}{\overset{\cdot }{\gamma }\eta_{m}}$$

where the component of K is negative for K < 1 and positive for K > 1. The average diameter (d) of the dispersed phase is dependent on the shear rate (γ), matrix viscosity (η_m_), interfacial tension (α), and the viscosity ratio of the two domains and it would increase with decreasing the matrix viscosity as a result of increasing temperature. The melting viscosity of the PLA matrix was much lower than that of EUG-*g*-GMA, which implied that K > 1 and the component of K was positive in the PLA/EUG-*g*-GMA blends. Therefore, it was presumed that the “sea-sea” phase structure discussed before was achieved at an optimum viscosity ratio because it was easier for EUG-*g*-GMA droplets to coalesce in the PLA matrix with pretty low melting viscosity at high temperature. It could also be explained by the truth that the melting strength of EUG-*g*-GMA was much higher than that of PLA matrix so it was not easy to break the rubber phase up and EUG-*g*-GMA remained in the continuous state even during dynamic vulcanization.

### 3.3. Improved Interface and Thermal Properties of the PLA/EUG-g-GMA TPVs

**Figure 5:**
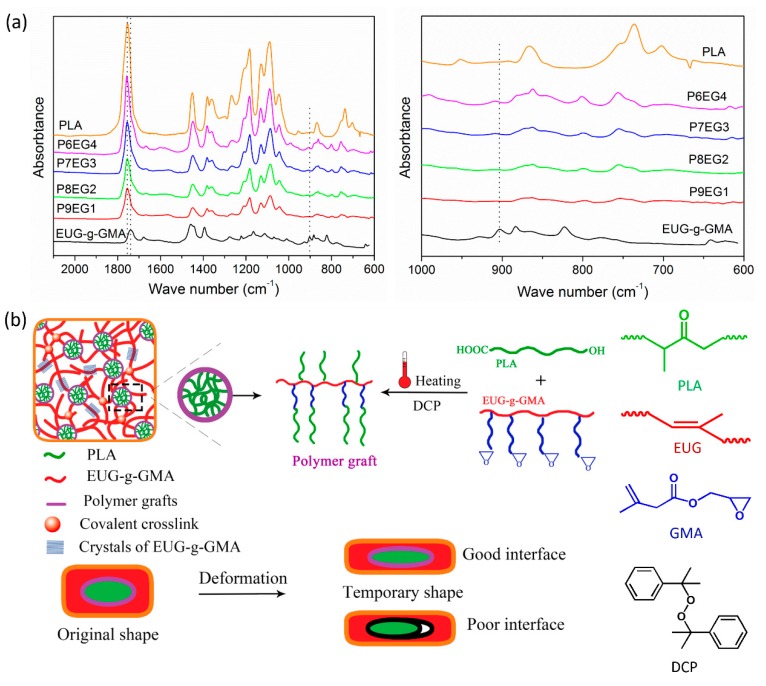
(**a**) FTIR spectra of PLA, EUG-*g*-GMA and DCM-extracted TPVs, (**b**) schematic diagram for in situ compatibilization in the PLA/EUG-*g*-GMA TPVs.

The mechanical properties and shape memory performances of TPVs were influenced by interfacial adhesion greatly. Therefore, FTIR was used to collect spectra of PLA, EUG-*g*-GMA and DCM-extracted PLA/EUG-*g*-GMA TPVs in order to study in situ compatibilization in the TPVs (Figure 5a). The sharp absorption peak at 1750 cm^−1^ in the spectrum of PLA belonged to carbonyl (C=O) as its characteristic absorption [28]. According to the previous discussion about the FTIR spectrum of the EUG-*g*-GMA, the absorption bands at 1725, 1250, and 908 cm^−1^ represented extension vibration of carbonyl (C=O) and epoxy groups [30], respectively. As expected, the spectra of the PLA/EUG-*g*-GMA TPVs were basically the same as that of EUG-*g*-GMA after extraction by DCM because free PLA was removed completely. However, the absorption peak of epoxy groups at 908 cm^−1^ disappeared, which indicated that epoxy groups had been reacted with C=O on PLA to form novel polymer grafts [30]. Furthermore, the absorption peak of C=O on the extracted residuals was observed at 1746 cm^−1^, showing a blue shift compared to pure EUG-*g*-GMA, which demonstrated the grafting reaction between PLA and EUG-*g*-GMA initiated by DCP during dynamic vulcanization [28]. These in situ compatibilization reactions improved the interfacial interaction between PLA and EUG-*g*-GMA largely.

Based on the FTIR analysis, a mechanism diagram for the interfacial compatibilization reaction was promoted (Figure 5b). In the early blending stage, epoxy groups on EUG-*g*-GMA reacted with carbonyl groups and hydroxyl groups on PLA to form copolymers. After the addition of DCP, two kinds of macromolecular radicals were formed via radical-seizing hydrogen atom. The two polymer radicals would interact with each other to form polymer grafts. The copolymers and polymer grafts could also be dynamically vulcanized to form the interfacial transition layer between the two parts so that interfacial adhesion in the TPVs was improved. The improved interface would provide assistance in keeping the elongation of the crosslinked EUG-*g*-GMA phase firmly during shape fixity and saving a strong restoring force to fulfill perfect shape recovery.

**Figure 6:**
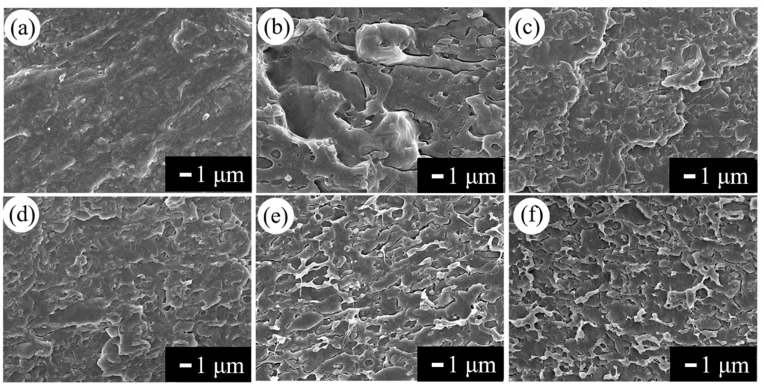
Fractured microstructure observed by SEM (**a**) neat PLA, (**b**) P6/E4 (**c**) P9EG1, (**d**) P8EG2, (**e**) P7EG3, and (**f**) P6EG4.

To further confirm the improved interface in PLA/EUG-*g*-GMA TPVs, fractured microstructure of the TPVs were observed. Neat PLA (Figure 6a) showed a typical brittle fractured surface, which was quite smooth with no plastic deformation. Though the PLA/EUG TPV (Figure 6b) showed a coarse fractured surface there was obvious phase separation in the blend, and EUG was dispersed in PLA matrix in large particles with a diameter of 5 µm, which resulted from poor compatibility between the two polymers. The coarser fractured surface was observed in the PLA/EUG-*g*-GMA TPVs (Figure 6c–f), and phase boundary was fuzzy, showing no “pulling out” phenomenon, which further proved the enhanced interfacial adhesion due to the in situ compatibilization.

**Figure 7:**
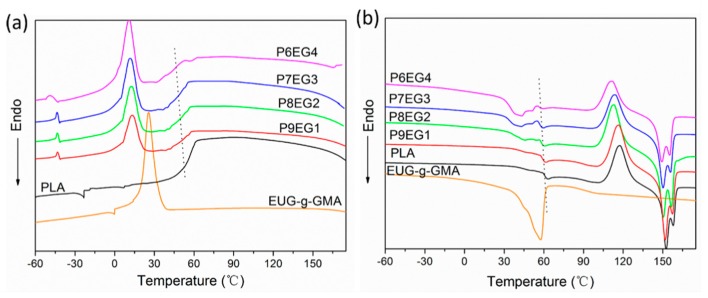
DSC curves of PLA, EUG-*g*-GMA and PLA/EUG-*g*-GMA TPVs (**a**) the first cooling scan, (**b**) the first heating scan.

DSC analysis was carried out to study the thermal properties of PLA/EUG-*g*-GMA TPVs. In the cooling scanning (Figure 7a), there was the glass transition of PLA at around 60 °C [36] and the crystallization peak of EUG-*g*-GMA ranging from −10 to 40 °C. For PLA/EUG-*g*-GMA TPVs, the glass transition temperature (*T*_g_) of PLA shifted to a lower temperature while the crystallization peak area of EUG-*g*-GMA increased obviously with increasing EUG-*g*-GMA content (indicated by dot line). In the heating scanning (Figure 7b), endothermic peak at around 50 °C corresponded to the melting of crystalline zones in the EUG-*g*-GMA phase [14]. Meanwhile, the PLA/EUG-*g*-GMA TPVs exhibited similar thermograms to pure PLA except for the crystallization melting of EUG-*g*-GMA, including glass transition, cold crystallization, and melting of crystalline regions in PLA. The melting peak of the EUG-*g*-GMA phase showed the same increasing trend in the area as the crystallization peak with an increase in the EUG-*g*-GMA content, but cold crystallization and crystallization melting peak of the PLA phase experienced a remarkable decrease in area, moving to a lower temperature like *T*_g_. It was believed that the “net-like” continuous EUG-*g*-GMA was throughout the TPVs, which reduced the molecular mobility of PLA. In addition, the molecular regularity of PLA molecules decreased as a result of in situ compatibilization, the grafting reaction of PLA to the EUG-*g*-GMA. Both of them contributed to the decrease in *T*_g_, crystallinity, and perfectness of the crystalline structure for the PLA phase. Besides, *T*_trans_ was set to 60 °C for shape memory analysis according to *T*_g_ of the PLA phase.

### 3.4. Shape Memory Analysis

**Figure 8:**
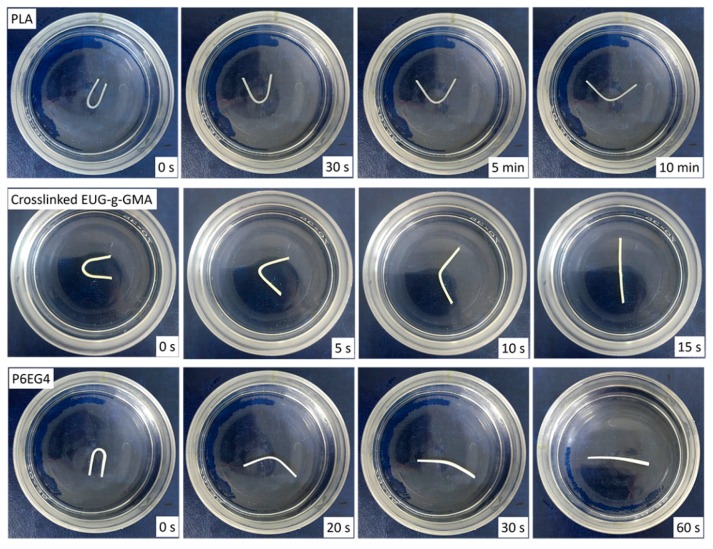
Shape recovery of crosslinked EUG-*g*-GMA, neat PLA and P6EG4 TPV at 60 °C.

Both PLA and partially crosslinked EUG-*g*-GMA possess shape memory capacity which was derived from the changes in entropy during glass transition and crystalline/melting phase transition, respectively. The PLA/EUG-*g*-GMA TPVs would exhibit excellent shape memory properties due to the co-continuous structure and improved interface. To describe HSME of the PLA/EUG-*g*-GMA TPVs induced by the two-phase transitions, shape recovery of neat PLA, crosslinked EUG-*g*-GMA and P6EG4 TPV at 60 °C for various times was recorded by a digital camera. As shown in Figure 8, although PLA could achieve shape recovery in 5 min the original linear shape could not be recovered after 10 min. However, crosslinked EUG-*g*-GMA returned to its original shape rapidly within 15 s. To our surprise, it just took 60 s for the P6EG4 TPV to fulfill the shape recovery from temporary shape to the permanent linear shape. The promoted shape recovery capacity might be attributed to the co-continuous phase structure and improved interfacial adhesion in the PLA/EUG-*g*-GMA TPVs.

**Figure 9:**
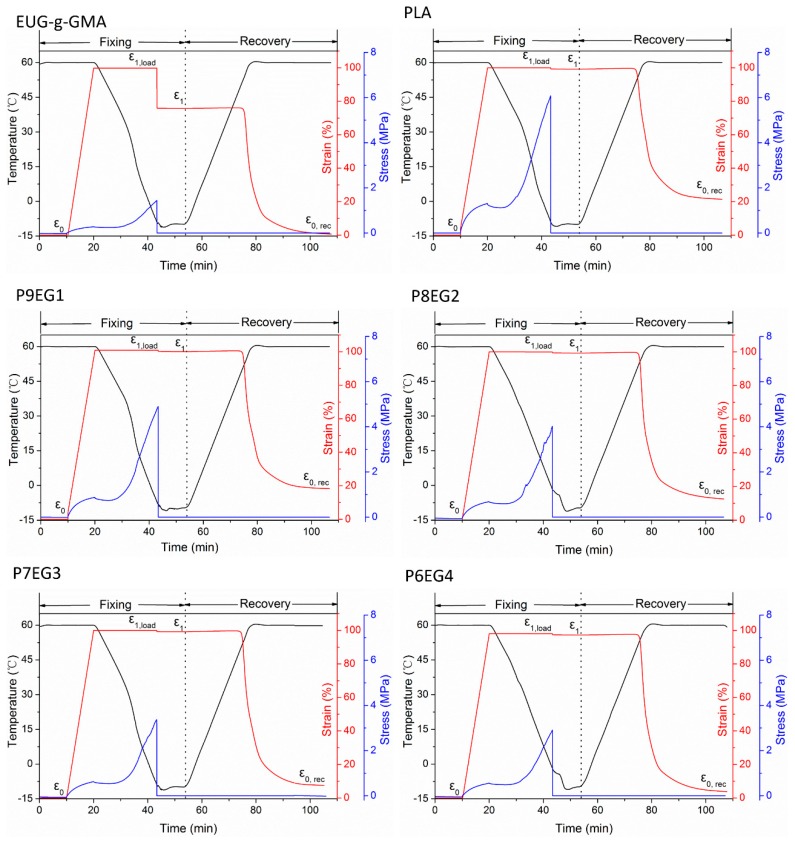
HSME of crosslinked EUG-*g*-GMA, neat PLA, and PLA/EUG-*g*-GMA TPVs with different EUG-g-GMA content.

To characterize the HSME quantitatively, strain-controlled tests were performed on a DMA analyzer, and results were shown in Figure 9. An external force was loaded to stretch the specimen to the temporary shape after being kept isothermally at 60 °C. Afterward, the shape was fixed by a cooling process followed by unloading. Finally, shape recovery was achieved upon reheating to 60 °C. Herein, parameters in Table 2, shape fixity ratio (*R_f_*) and shape recovery ratio (*R_r_*), were defined to measure the shape memory properties. Crosslinked EUG-*g*-GMA displayed worse shape fixing ability with an *R_f_* of 77.34%. However, neat PLA and the TPVs exhibited pretty high shape fixing capacity due to the restriction from rigid PLA and crystalline regions of EUG-*g*-GMA at −10 °C, giving a shape fixity ratio (*R_f_*) of nearly 100%. The TPV with larger EUG-*g*-GMA content displayed better shape recovery ability, and the maximum shape recovery ratio (*R_r_*) of 93.74% was reached at 40 wt % EUG-*g*-GMA content. Shape recovery of pure PLA and strong entropy resilience of EUG-*g*-GMA were two main contributors for the excellent shape recovery in the TPVs [28]: (1) PLA showed a moderate *R_f_* of 70.47% to demonstrate the inherent mild recovery of the PLA phase in the TPVs. The frozen PLA molecules were in an entropic unstable state at −10 °C and they were disorientated once reheating to 60 °C so that the restricted crosslinked EUG-*g*-GMA phase was released to drive shape recovery. (2) During the shape fixity process, crystalline zones of EUG-*g*-GMA worked together with rigid PLA molecules to hold the temporary shape, and strong entropy resilience was stored to the “netlike” crosslinked EUG-*g*-GMA phase in an amorphous state. When the crystals were melted at 60 °C, the rubber phase experienced instantaneous retraction due to the entropic resilience, leading to high *R_r_*. *R_f_* of crosslinked EUG-*g*-GMA was 99.48%. Therefore, there was no doubt that the TPV with higher EUG-*g*-GMA content had better shape recovery capacity because the strength of the rubber phase was enhanced.

**Figure 10:**
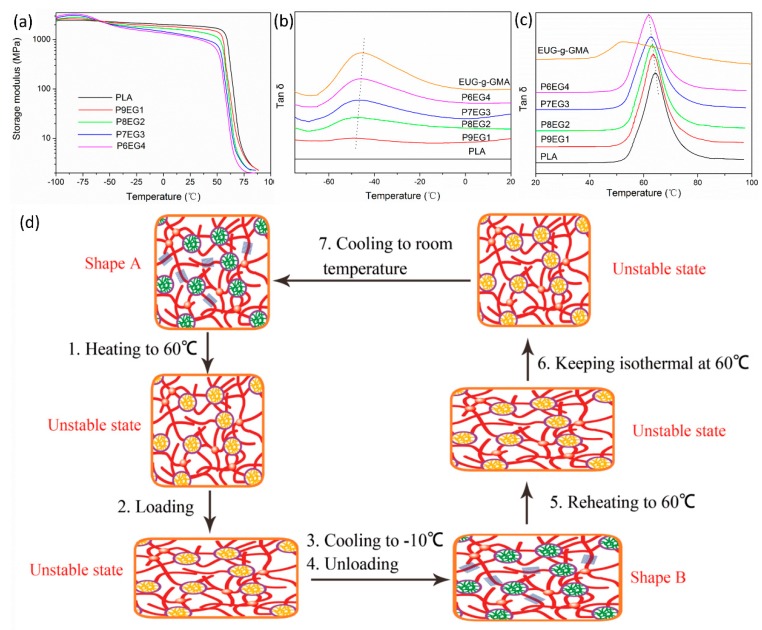
(**a**) Storage modulus-temperature curves of PLA and PLA/EUG-*g*-GMA TPVs, (**b**) loss factor-temperature curves ranging from −85 to 20 °C, (**c**) loss factor-temperature curves ranging from 20 to 100 °C, (**d**) schematic diagram of shape memory mechanism.

To further discuss the shape memory mechanism, temperature sweep was performed on a DMTA analyzer for neat PLA and the PLA/EUG-*g*-GMA TPVs, shown in Figure 10a. The decline in storage modulus (E’) of neat PLA at around 65 °C was induced by its glass transition, however, two occurred in the TPVs. One at a lower temperature (~−50 °C) was attributed to the glass transition of EUG-*g*-GMA [37], while the other (~65 °C) had a close relationship with a glass transition of PLA [32,38] and melting of EUG-*g*-GMA crystals [14]. The crosslinked EUG-*g*-GMA phase was in the consecutive state throughout the TPVs, and it acted as hard “backbones” in the frozen TPVs at −90 °C, which generated a higher E’ than pure PLA, benefiting shape recovery. However, the TPVs exhibited lower E’ in the temperature range from −40 to 50 °C because EUG-*g*-GMA was soft with large elasticity after glass transition. Even so, E’ of the TPVs at −40~50 °C was almost 1000 times than that at above 65 °C, which suggested that E’ originated from the frozen PLA and EUG-*g*-GMA crystals was far beyond the entropic elasticity in the stretched rubber phase to hold the temporary shape firmly, resulting in high *R_f_*. When frozen PLA molecules and EUG-*g*-GMA molecular segments in crystals regained their mobility, E’ decreased sharply. At the same time, resilience stored in the netlike EUG-*g*-GMA phase worked as a restoring force to drive shape recovery of the TPVs.

Glass transition temperature (*T*_g_) of crosslinked EUG-*g*-GMA was at around −50 °C in Figure 10b. EUG-*g*-GMA phase in the TPVs displayed a slight lower *T*_g_ than crosslinked EUG-*g*-GMA, shifting from −49.6 to −47.1 °C as EUG-*g*-GMA content increased from 10 wt % to 40 wt % (indicated by dot lines). The tan δ peaks at ~65 °C in Figure 10c represented the *T*_g_ of PLA in which onset temperatures were located at 51–53 °C, where the E’ began to decline again. In other word, amorphous PLA molecular chains and segments began to move at 51–53 °C, so 60 °C might be an ideal T_trans_ for HSME of the TPVs. In addition, *T*_g_ of the PLA phase decreased from 64.7 to 61.5 °C over the EUG-*g*-GMA content range from 10 wt % to 40 wt %. The changes of *T*_g_ in both the EUG-*g*-GMA phase and the PLA phase further confirmed the effective interfacial compatibilization.

A schematic diagram in Figure 10c was also promoted to explain HSME of the dynamically vulcanized PLA/EUG-*g*-GMA TPVs. At low temperatures, such as −10 °C, PLA was in the glassy state and EUG-*g*-GMA crystals were present, shown by green lines and blue squares, respectively. Above *T*_trans_ (60 °C), molecular segments and chains of PLA and EUG-*g*-GMA began to move so that the sample was deformed into arbitrary shape easily. Next, the temporary shape was fixed by cooling to a temperature below *T*_trans_, in which orientation of PLA and elongation of crosslinked EUG-*g*-GMA network was frozen, meanwhile restoring force was saved. The specimen underwent no shrinkage after releasing external force due to the restriction from the EUG-*g*-GMA crystals and the rigid PLA with continuous structure. As the sample was reheated to *T*_trans_, disorientation of PLA molecules and melting of EUG-*g*-GMA crystalline regions made the crosslinked rubber domain “unlocked”. Strong entropy resilience stored in the rubber network and disorientation worked in conjunction to realize perfect shape recovery. The co-continuous architecture played a critical role in the improved shape memory behaviors of the TPVs.

### 3.5. Mechanical Characterization

**Figure 11:**
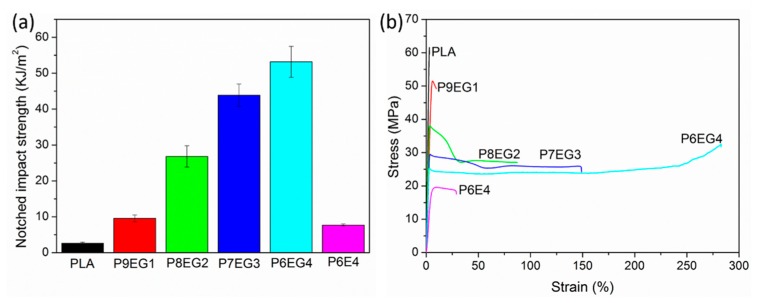
Mechanical properties of neat PLA, PLA/EUG-*g*-GMA TPVs, and PLA/EUG TPV (**a**) notched impact strength, (**b**) stress-strain curves.

Mechanical properties are shown in Figure 11. We found that neat PLA exhibited high brittleness, giving a notched impact strength of 2.4 kJ/m^2^ in Figure 11a, which was consistent with its tensile performance with tensile strength and elongation at break of 67.8 MPa and 4.7% according to stress-strain curves in Figure 11b. However, the PLA/EUG-*g*-GMA TPVs showed enhanced toughness. Although the yielding strength of the TPVs experienced investable sacrifice with increasing EUG-*g*-GMA content, both elongation at break and impact strength increased abruptly. Moreover, the tensile yielding behavior of the TPVs tended to become perfect, which was dominated by the promoted interface [30,39] and the “sea-sea” phase structure. A super toughened PLA based TPV was obtained at EUG-*g*-GMA content of 40 wt %, whose elongation at break was 285% and impact strength was 54.8 kJ/m^2^, seven times more than that of P6E4 TPV (7.6 kJ/m^2^) and 20 times more than that of PLA (2.4 kJ/m^2^).

## 4. Conclusions

This work introduced an efficient and green modification method for natural EUG to improve its polarity via the bulk radical grafting reaction of GMA monomer. The modified EUG (EUG-*g*-GMA) was compatible with PLA, and a novel fully biobased shape memory TPVs were fabricated based on the two biopolymers using a dynamic vulcanization technique. The PLA/EUG-*g*-GMA TPVs presented a co-continuous phase structure and improved interface, which were the main contributors to enhancement in shape memory capacity and toughness. The TPV exhibited the best shape memory properties at EUG-*g*-GMA content of 40 wt %, excellent shape fixing capacity (*R_f_* = 99.83%), and highest shape recovery ratio (*R_r_* = 93.74%). These shape memory TPVs would provide a new approach to produce biobased shape memory materials applied in intelligent medical devices.

## Figures and Tables

**Table 1:** Formulas of PLA/EUG-*g*-GMA TPVs.

Chemicals	Weight Ratio					
	Crosslinked EUG-*g*-GMA	P9EG1	P8EG2	P7EG3	P6EG4	P6E4
PLA	0	90	80	70	60	60
EUG-*g*-GMA	100	10	20	30	40	0
EUG	0	0	0	0	0	40
Antioxidant1010	0.2	0.2	0.2	0.2	0.2	0.2
DCP	0.15	0.15	0.3	0.45	0.6	0.6

**Table 2:** Shape memory performances of crosslinked EUG-*g*-GMA, PLA, and PLA/EUG-*g*-GMA TPVs.

Properties	Crosslinked EUG-*g*-GMA	Neat PLA	P9EG1	P8EG2	P7EG3	P6EG4
*R_f_* (%)	77.34	99.54	99.64	99.63	99.63	99.83
*R_r_* (%)	99.48	70.47	83.28	86.57	90.97	93.74
